# Salinity Tolerance of Halophytic Grass *Puccinellia nuttalliana* Is Associated with Enhancement of Aquaporin-Mediated Water Transport by Sodium

**DOI:** 10.3390/ijms23105732

**Published:** 2022-05-20

**Authors:** Maryamsadat Vaziriyeganeh, Micaela Carvajal, Ning Du, Janusz J. Zwiazek

**Affiliations:** 1 Department of Renewable Resources, University of Alberta, Edmonton, AB T6G 2E3, Canada; vaziriye@ualberta.ca; 2 Group of Aquaporins, Plant Nutrition Department, Centre of Edaphology and Applied Biology of Segura (CEBAS-CSIC), Campus of Espinardo, Building 25, E-30100 Murcia, Spain; mcarvaja@cebas.csic.es; 3 Institute of Ecology and Biodiversity, School of Life Science, Shandong University, Qingdao 266237, China; ndu@sdu.edu.cn

**Keywords:** aquaporins, cell hydraulic conductivity, gas exchange, halophytes, sodium, water relations

## Abstract

In salt-sensitive plants, root hydraulic conductivity is severely inhibited by NaCl, rapidly leading to the loss of water balance. However, halophytic plants appear to effectively control plant water flow under salinity conditions. In this study, we tested the hypothesis that Na^+^ is the principal salt factor responsible for the enhancement of aquaporin-mediated water transport in the roots of halophytic grasses, and this enhancement plays a significant role in the maintenance of water balance, gas exchange, and the growth of halophytic plants exposed to salinity. We examined the effects of treatments with 150 mM of NaCl, KCl, and Na_2_SO_4_ to separate the factors that affect water relations and, consequently, physiological and growth responses in three related grass species varying in salt tolerance. The grasses included relatively salt-sensitive *Poa pratensis*, moderately salt-tolerant *Poa juncifolia*, and the salt-loving halophytic grass *Puccinellia nuttalliana*. Our study demonstrated that sustained growth, chlorophyll concentrations, gas exchange, and water transport in *Puccinellia nuttalliana* were associated with the presence of Na in the applied salt treatments. Contrary to the other examined grasses, the root cell hydraulic conductivity in *Puccinellia nuttalliana* was enhanced by the 150 mM NaCl and 150 mM Na_2_SO_4_ treatments. This enhancement was abolished by the 50 µM HgCl_2_ treatment, demonstrating that Na was the factor responsible for the increase in mercury-sensitive, aquaporin-mediated water transport. The observed increases in root Ca and K concentrations likely played a role in the transcriptional and (or) posttranslational regulation of aquaporins that enhanced root water transport capacity in *Puccinellia nuttalliana*. The study demonstrates that Na plays a key role in the aquaporin-mediated root water transport of the halophytic grass *Puccinellia nuttalliana*, contributing to its salinity tolerance.

## 1. Introduction

Salinity is among the most challenging problems faced by terrestrial plants in many parts of the globe due to the accumulation of salts in the soil through natural processes and human activities. Although soil salinity may refer to the presence of elevated concentrations of different salts, sodium salts, especially when accompanied by chloride, are the most common and detrimental salts affecting plants worldwide [1]. However, sulphates, carbonates and bicarbonates, as well as calcium, magnesium and potassium ions, often contribute to soil salinity [2].

The majority of terrestrial plants are salt-sensitive glycophytes and only about 2% of plants have been classified as halophytes that can tolerate high salt concentrations [3]. Although salt tolerance levels widely vary between glycophytes, these plants have evolved by adapting to soils with low soil Na levels and maintain low Na concentrations in their aboveground tissues [4]. Halophytic plants also vary in their level of salt tolerance and in one of the commonly used classifications, these salt tolerant plants are divided into obligate, facultative, and habitat indifferent halophytes [5]. Obligate halophytes (euhalophytes) require salt for their survival and usually show the optimum growth and development in NaCl concentrations exceeding 200 mM [3]. In contrast, facultative halophytes can survive without salt, but their growth is enhanced by moderate salinity and is reduced by both low and high salt concentrations. The habitat indifferent halophytes are plants that prefer salt-free soils but can also cope with relatively high salt concentrations [6].

The salt tolerance of halophytes has attracted considerable attention and many processes contributing to salt tolerance have been described for various species of halophytic plants. However, some of the fundamental aspects of salt tolerance in halophytes related to water relations remain obscure. The ability of plants to cope with salinity is largely determined by their ability to maintain the acquisition of water and mineral nutrients and to protect their tissues against direct ion toxicity, oxidative stress, and osmotic effects of salts [7,8]. The maintenance of plant water balance requires complex and precise control and coordination of the processes of water uptake, water movement within the plant, and water loss. Water flow in most plants encounters the most resistance when crossing the root tissues between the epidermis and the root xylem [9]. A sharp increase in the root water flow resistance (decreased root hydraulic conductivity) is among the earliest responses to salt observed in glycophytes [10,11,12]. The root water flow resistance is dynamically controlled by root aquaporins which are an integral part of the cell-to-cell pathway [13] and are sensitive to NaCl [14,15,16]. The decrease in root hydraulic conductivity triggers stomatal closure and a decrease in transpiration rates [17], which reduces the relative contribution of hydrostatic forces and increases the significance of osmotic forces that are altered by salt.

In salt-sensitive plants, the inhibition of aquaporin function by salt is rapid and strong [16,18,19]. The aquaporin-mediated root water transport and cell hydraulic conductivity were inhibited by about three-fold in *Arabidopsis* within several minutes, following root exposure to concentrations of NaCl as low as 10 mM [16]. However, in the halophytic grass *Puccinellia nuttalliana*, cell hydraulic conductivity was enhanced by the treatments with 50 and 150 mM NaCl, leading to the hypothesis that NaCl may enhance the aquaporin-mediated transport in roots of halophytic plants [20]. A subsequent study demonstrated that the six-day treatment with 150 mM NaCl triggered an increase in the gene expression of *PnPIP2;2*, suggesting that this aquaporin in *Puccinellia nuttalliana* may be key to maintaining efficient root water transport under salinity conditions [21].

Salt affects plants through a combination of osmotic, ionic, nutritional, and oxidative factors [1,7,22], which can potentially alter plant water transport and water relations [23]. Amelioration of root hydraulic conductivity [14], root cell hydraulic conductivity [16], and osmotic water permeability in plasma membrane vesicles [15] by Ca^2+^ in NaCl-treated plants point to a direct ion effect on the aquaporin-mediated water transport. However, treatments of barley (*Hordeum vulgare*) with the same osmolarity solutions of NaCl and sorbitol had an almost identical effect on root hydraulic conductivity [24]. Salinity tolerance of halophytic plants is often attributed to their efficient Na management [3]. NaCl secretion through salt glands by some halophytes and the more efficient compartmentalization of Na compared with K in the vacuoles [25,26,27] may partly explain the greater toxicity of high treatment concentrations with K salts compared with similar concentrations of NaCl. However, the difference in ion management does not fully explain the growth enhancement by NaCl that has also been reported for some halophytic plants [20,25].

Since the exact signals triggering an enhancement of root hydraulic conductivity in *Puccinellia nuttalliana* by NaCl [20] are unclear, the present study was designed to separate different ionic factors in their effects on the root water transport properties and physiological processes in three related northern grass species varying in salt tolerance. The grasses included relatively salt-sensitive Kentucky bluegrass (*Poa pratensis*), moderately salt-tolerant alkali bluegrass (*Poa juncifolia*), and the salt-loving halophytic Nuttall’s alkaligrass (*Puccinellia nuttalliana*) [20,28]. Plants were treated for up to 10 days with 150 mM NaCl, 150 mM KCl, and 150 mM Na_2_SO_4_ and their growth and physiological responses, including water relations and the aquaporin-mediated cell-to-cell root water transport, were examined. We tested the hypothesis that Na^+^ is the principal factor responsible for the enhancement of cell hydraulic conductivity in the roots of halophytic grasses and this enhancement plays a significant role in the maintenance of water balance, gas exchange, and the growth of halophytic plants exposed to salinity.

## 2. Results

### 2.1. Plant Morphology and Dry Weights (DW)

Stunted roots and shoots, as well as extensive leaf chlorotic and leaf necrotic lesions, were observed in *Poa pratensis* plants after 10 days of treatments with 150 mM NaCl, 150 mM Na_2_SO_4_, and 150 mM KCl (Supplementary Figure S1). The control plants of *Puccinellia nuttalliana* and the plants treated with 150 mM KCl exhibited leaf chlorosis that was not observed in plants treated with 150 mM NaCl and 150 mM Na_2_SO_4_ (Supplementary Figure S1).

**Figure 1:**
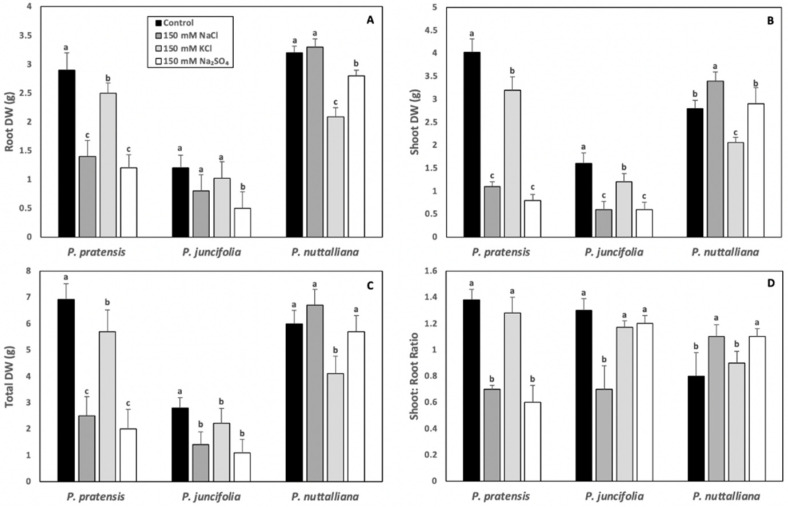
Root (**A**), shoot (**B**), and total dry weights (DW) (**C**) and shoot to root DW ratios (**D**) in *Poa pratensis*, *Poa juncifolia* and *Puccinellia nuttalliana* treated for 10 days with 150 mM NaCl, 150 mM KCl, 150 mM Na_2_SO_4_ and in control (untreated) plants. Different letters or numbers above the bars indicate significant differences (*p* ≤ 0.05) between treatments within each species as determined by the Tukey’s test. Means (*n* = 6) and SE are shown.

Root, shoot, and total DW of *Poa pratensis* were sharply reduced in plants treated for 10 days with 150 mM NaCl and 150 mM Na_2_SO_4_ and these reductions were several-fold greater compared with the plants treated with 150 mM KCl (Figure 1A–C). The shoot: root DW ratios were reduced by the 150 mM NaCl and Na_2_SO_4_ treatments but were not changed in plants treated with 150 mM KCl (Figure 1D).

The root DW in *Poa juncifolia* was significantly reduced by the 150 mM Na_2_SO_4_ treatment (Figure 1A). The shoot (Figure 1B) and total (Figure 1C) DW were significantly decreased by all salt treatments, but the reductions were greater in plants treated with the Na salts compared with KCl. Only 150 mM NaCl significantly reduced shoot: root DW ratios in *Poa juncifolia* (Figure 1D).

In *Puccinellia nuttalliana*, the root DW was not affected by 150 mM NaCl but was reduced by the 150 mM KCl and 150 mM Na_2_SO_4_ treatments compared with the untreated control (Figure 1A). A reduction in shoot DW was observed in plants treated with 150 mM KCl, while it was enhanced by 150 mM NaCl (Figure 1B). There was no effect of 150 mM Na_2_SO_4_ treatment on the shoot DW (Figure 1B). The total DW was reduced in *Puccinellia nuttalliana* by the 150 mM KCl treatment (Figure 1C), while the shoot: root DW ratios were enhanced by the 150 mM NaCl and 150 mM Na_2_SO_4_ treatments (Figure 1D).

### 2.2. Net Photosynthesis (Pn) and Transpiration (E) Rates

**Figure 2:**
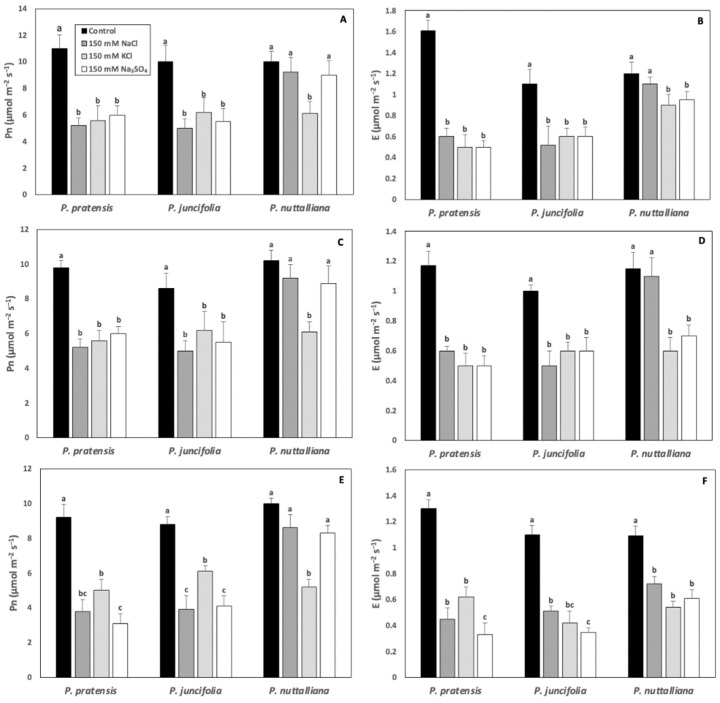
Net photosynthesis (Pn) (**A**,**C**,**E**) and transpiration (**E**). (**B**,**D**,**F**) rates in *Poa pratensis*, *Poa juncifolia*, and *Puccinellia nuttalliana* after three (**A**,**B**), six (**C**,**D**) and nine (**E**,**F**) days of treatments with 150 mM NaCl, 150 mM Na_2_SO_4_, 150 mM KCl, and in untreated control plants. Different letters above the bars indicate significant differences (*p* ≤ 0.05) between treatments within each species as determined by the Tukey’s test. Means (*n* = 6) and SE are shown.

Compared with control plants, Pn was sharply reduced in *Poa pratensis* and *Poa juncifolia* by all salt treatments after 3, 6, and 9 days. (Figure 2A,C,E). In *Puccinellia nuttalliana*, there was no effect of 150 NaCl and 150 Na_2_SO_4_ on Pn. However, Pn declined compared with control in plants treated with 150 mM KCl on all measurement days (Figure 2A,C,E).

In *Poa pratensis* and *Poa juncifolia*, E decreased compared with their respective controls in all salt treatments and on all treatment days (Figure 2B,D,F). In *Puccinellia nuttalliana*, after 3 and 6 days of treatments, E decreased in plants treated with 150 mM KCl and 150 mM Na_2_SO_4_, but not with 150 mM NaCl (Figure 2B,D). After 9 days, all salt treatments resulted in a significant decrease in E in *Puccinellia nuttalliana* (Figure 2F).

### 2.3. Leaf Chlorophyll Concentrations

**Figure 3:**
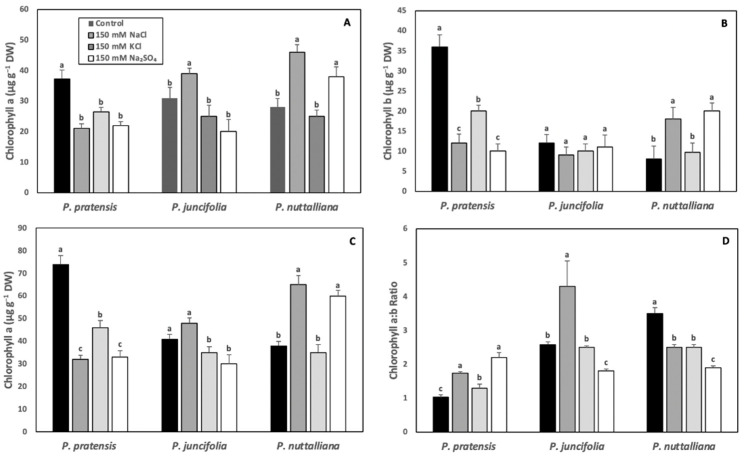
Leaf chlorophyll a (**A**), chlorophyll b (**B**), and total chlorophyll (**C**) concentrations, and chlorophyll a:b ratios (**D**) in *Poa pratensis*, *Poa juncifolia*, and *Puccinellia nuttalliana* subjected to six days of treatments with 150 mM NaCl, 150 mM KCl, 150 mM Na_2_SO_4_ and in untreated control plants. Different letters above the bars indicate significant differences (*p* ≤ 0.05) between treatments within each species as determined by the Tukey’s test. Means (*n* = 6) and SE are shown.

After six days of treatments, chlorophyll a, chlorophyll b, and total leaf chlorophyll concentrations decreased and chlorophyll a:b ratios increased in *Poa pratensis* exposed to 150 mM NaCl, 150 mM KCl and 150 mM Na_2_SO_4_ compared with the control plants (Figure 3A–D).

In *Poa juncifolia*, leaf chlorophyll a concentration was significantly increased by the 150 mM KCl treatment (Figure 3A). There was no significant treatment effect on the chlorophyll b concentration (Figure 3B). The total chlorophyll concentration was unchanged in the 150 mM NaCl treatment and decreased in plants treated with 150 mM KCl and 150 mM Na_2_SO_4_ (Figure 3C). The chlorophyll a:b ratios increased in the 150 mM NaCl treatment and decreased in the 150 mM Na_2_SO_4_ treatment (Figure 3D).

In *Puccinellia nuttalliana*, there was a large increase in the leaf chlorophyll a, chlorophyll b, and total chlorophyll concentrations in plants treated with 150 mM NaCl and 150 mM Na_2_SO_4_, but there was no effect of KCl on the leaf chlorophyll concentrations (Figure 3A–C). The leaf chlorophyll a:b ratios decreased in *Puccinellia nuttalliana* in all salt treatments (Figure 3D).

### 2.4. Leaf Water Potentials (ψ_w_) and Shoot Water Contents (WC)

**Figure 4:**
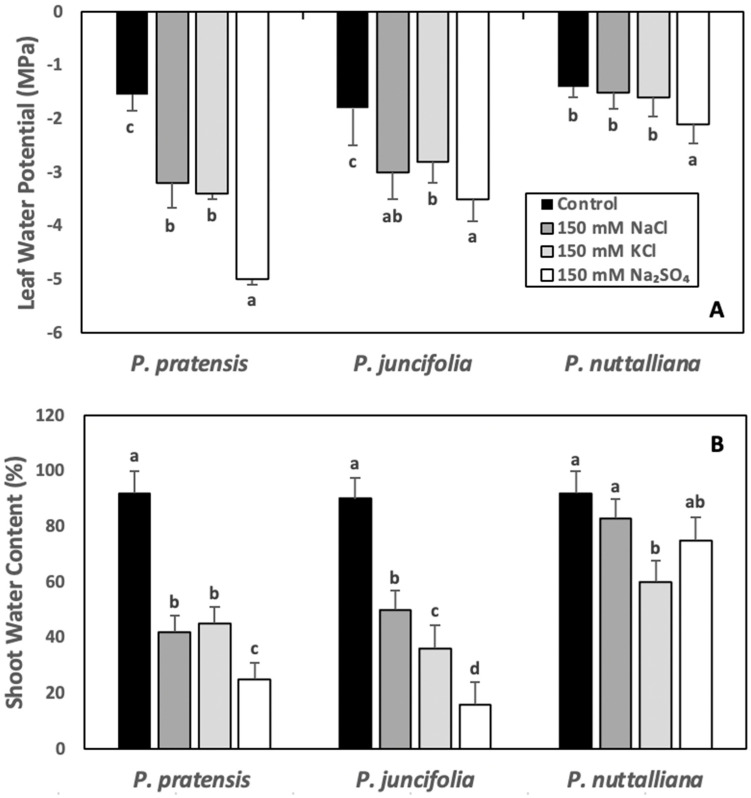
Leaf water potentials (**A**) and shoot water contents (**B**) in *Poa pratensis*, *Poa juncifolia*, and *Puccinellia nuttalliana* treated with 150 mM NaCl, 150 mM KCl, and 150 mM Na_2_SO_4_ and in untreated control plants. The measurements of leaf water potentials were carried out after six days and shoot water potentials after ten days of treatments. Different letters above the bars indicate significant differences (*p* ≤ 0.05) between treatments within each as determined by the Tukey’s test. Means (*n* = 6) and SE are shown.

After six days of 150 mM NaCl, 150 mM KCl, and 150 Na_2_SO_4_ treatments, ψ_w_ decreased in *Poa pratensis* and *Poa juncifolia* and the greatest decrease was observed in plants treated with 150 mM Na_2_SO_4_ (Figure 4A). In *Puccinellia nuttalliana*, there was no significant effect of NaCl and KCl treatments on ψ_w_, but the ψ_w_ values were slightly lower in plants treated with 150 mM Na_2_SO_4_ (Figure 4A).

The shoot WC decreased in *Poa pratensis* and *Poa juncifolia* subjected to six days of 150 mM NaCl, 150 mM KCl, and 150 mM Na_2_SO_4_ treatments compared with untreated control and the greatest decrease was measured in plants treated with 150 mM Na_2_SO_4_ (Figure 4B). In *Puccinellia nuttalliana*, WC of plants treated with NaCl and Na_2_SO_4_ was similar to the untreated control plants; however, WC was significantly reduced by the 150 mM KCl treatment compared with the untreated control (Figure 4B).

### 2.5. Cell Hydraulic Conductivity (L_pc_)

**Figure 5:**
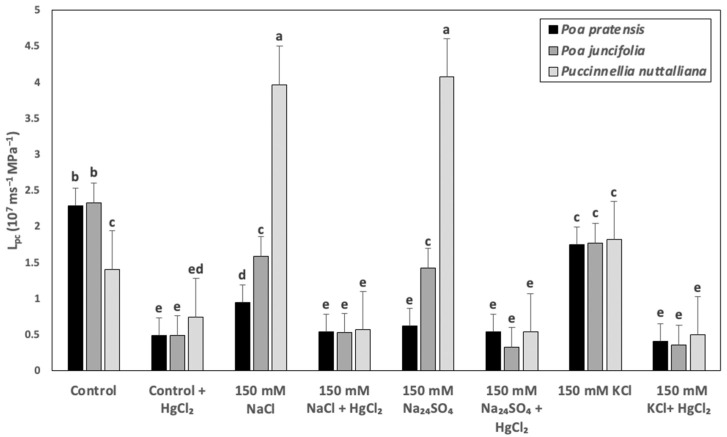
Cell hydraulic conductivity (L_pc_) of root cortex cells in *Poa pratensis*, *Poa juncifolia*, and *Puccinellia nuttalliana* plants treated with 150 mM NaCl, 150 mM Na_2_SO_4_, and 150 mM KCl for 6 days and in untreated control. The roots were exposed to their respective salt treatments in 50% Hoagland’s solutions (no salt for controls) followed by 50 µM HgCl_2_. Means (*n* = 6) and SE are shown. Different letters above the bars indicate significant differences (*p* ≤ 0.05) between treatments and species as determined by the Tukey’s test.

The L_pc_ in *Poa pratensis* treated with 150 mM NaCl and 150 mM Na_2_SO_4_ decreased by two- to three-fold and only a relatively smaller decrease was measured in plants treated with 150 mM KCl compared with control plants (Figure 5). Lower magnitude decreases of L_pc_ compared with *Poa pratensis* were also observed in the roots of *Poa juncifolia* treated with 150 mM NaCl, 150 mM Na_2_SO_4_, and 150 mM KCl (Figure 5). In *Puccinellia nuttalliana*, the L_pc_ values increased by approximately three-fold in the 150 mM NaCl and 150 mM Na_2_SO_4_ treatments and were not affected by 150 mM KCl (Figure 5). Treatments with 50 µM HgCl_2_ decreased the L_pc_ values to similar levels in all plant species, regardless of the treatment (Figure 5).

### 2.6. Root and Shoot Elemental Concentrations

**Figure 6:**
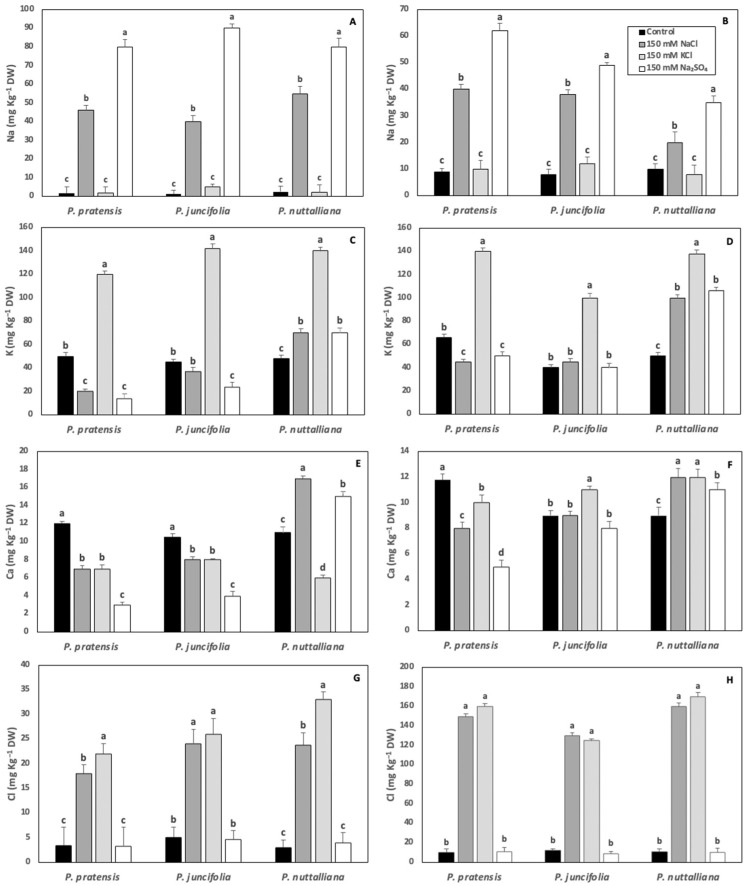
Concentrations of Na, K, Ca, and Cl in roots (**A**,**C**,**E**,**G**) and shoots (**B**,**D**,**F**,**H**) of *Poa pratensis*, *Poa juncifolia*, and *Puccinellia nuttalliana* after 10 days of treatments with 150 mM NaCl, 150 KCl, 150 mM Na_2_SO_4_ and in untreated control plants. Different letters above the bars indicate significant differences (*p* ≤ 0.05) between treatments within each plant species as determined by the Tukey’s test. Means (*n* = 6) and SE are shown.

Root Na concentrations increased to similar levels in the three plant species after ten days of 150 mM NaCl and 150 mM Na_2_SO_4_ treatments (Figure 6A). Shoot Na concentrations increased by approximately the same magnitude in *Poa pratensis* and *Poa juncifolia* treated with 150 mM NaCl. However, the Na shoot concentrations in *Puccinellia nuttalliana* treated with 150 mM NaCl was less than a half of the concentrations measured in the two other plant species (Figure 6B). The shoot Na concentrations in all three species of plants treated with 150 mM Na_2_SO_4_ were higher compared with the plants treated with 150 mM NaCl (Figure 6B).

The concentrations of K in roots and shoots of all plant species increased by about three-fold compared with the untreated control as a result of the 150 mM KCl treatment (Figure 6C,D). Both the 150 mM NaCl and 150 mM Na_2_SO_4_ treatments decreased root and shoot K concentrations in *Poa pratensis*. There was no effect of 150 mM NaCl on the K root and shoot concentrations and no effect of 150 mM Na_2_SO_4_ on the K shoot concentrations in *Poa juncifolia* (Figure 6C,D). However, the 150 mM Na_2_SO_4_ treatment decreased K root concentrations in *Poa juncifolia* compared with control plants (Figure 6C). In *Puccinellia nuttalliana* treated with 150 mM NaCl and 150 mM Na_2_SO_4_, root and shoot K concentrations increased compared with the untreated control and the increase was especially pronounced in shoots (Figure 6C,D).

All three salt treatments decreased root and shoot Ca concentrations in *Poa pratensis* (Figure 6E,F). In *Poa juncifolia*, root Ca concentrations were also reduced by all salt treatments (Figure 6E). However, there was no significant effect of 150 mM NaCl and 150 mM Na_2_SO_4_ and a small increase in Ca shoot concentration as a result of the 150 mM KCl treatment (Figure 6F). The 150 mM KCl treatment decreased Ca root concentrations and increased Ca shoot concentrations in *Puccinellia nuttalliana* (Figure 6E,F). Both 150 mM NaCl and 150 mM Na_2_SO_4_ triggered increases in root and shoot Ca concentrations in *Puccinellia nuttalliana* (Figure 6E,F).

Root Cl concentrations increased by several-fold in all three species of plants subjected to 150 mM NaCl and 150 mM KCl treatments (Figure 6G). The shoot Cl concentrations sharply increased in the three examined plant species treated with 150 mM NaCl and 150 mM KCl compared with the control and the concentrations were higher compared with the roots in the same treatments (Figure 6H).

## 3. Discussion

In the present study, we aimed at understanding how the different salt factors affect root water transport properties and physiological responses in three species of northern grasses varying in salt tolerance. Salinity stress can be caused by various forms of salts, which trigger a complex array of structural and functional responses that enable plants to avoid and tolerate the consequences of osmotic imbalance, oxidative stress, and ion toxicity [29]. Salt-induced water deficit stress is a common response in glycophytic plants and is responsible for the stomatal closure that results in decreases in gas exchange parameters and, in the longer-term, by growth inhibition, tissue necrosis, and plant mortality [30]. Signs of visible injuries were apparent in the glycophytic grass *Poa pratensis* in all salt treatments and the effects of these treatments on *Poa juncifolia* included largely stunted growth. The magnitude of these growth reductions was greater in *Poa pratensis* compared with a moderately salt tolerant *Poa juncifolia* which, similarly to the earlier study [20], showed slow growth compared with the other two plant species, also under control conditions. A different pattern was observed in the halophytic *Puccinellia nuttalliana* plants, which were visibly larger in the two Na-salt treatments compared with control plants and the plants treated with KCl. These observations were corroborated by the plant dry biomass measurements, which demonstrated no effects of 150 mM NaCl and 150 mM Na_2_SO_4_ on the total dry weight of *Puccinellia nuttalliana* and the reduction in the total dry weight by the 150 mM KCl treatment. Although Na is considered to be the main cause of ion toxicity in salt-sensitive plants [7], deleterious effects of salinity also involve osmotic factors as well as accompanying anions [22]. Since osmotic potentials of 150 mM NaCl are by about one-third higher (less negative) compared with 150 mM Na_2_SO_4_, and NaCl and KCl contain the same Cl^−^ concentrations, our results point to Na as a likely direct factor contributing to salinity tolerance in *Puccinellia nuttalliana* and the main detrimental factor to *Poa pratensis*.

Sodium salts affected shoot dry weights in *Poa pratensis* and *Poa juncifolia* more than root dry weights, resulting in a decrease in shoot: root DW ratios, while an increase in shoot: root DW ratios was observed in *Puccinellia nuttalliana*. A decrease in the shoot to root ratios is a common response to salinity in salt-sensitive plants and was proposed to be the consequence of the osmotic effect rather than the ion toxicity [31]. However, in our study, only NaCl and Na_2_SO_4_, and not KCl, significantly reduced shoot to root ratios in *Poa pratensis*, pointing to ion toxicity as the principal factor contributing to changes in growth allocations, as also reported for soybean [32].

Both Pn and E significantly decreased in *Poa pratensis* and *Poa juncifolia* after 3, 6, and 9 days of treatments with 150 mM NaCl, 150 mM KCl, and 150 mM Na_2_SO_4_. However, only the 150 mM KCl treatment inhibited Pn in *Puccinellia nuttalliana* despite the reductions in E by all salts after 9 days of treatments. The decreases in Pn and E in all three plant species treated with 150 mM KCl suggest little differences between halophytic and glycophytic grasses in their abilities to cope with K excess. Potassium is the principal ion used to control osmotic balance and stomatal opening [33]. However, some halophytic plants can substitute K with Na to promote stomatal opening since the availability of K may be affected by the salinity conditions [34,35]. Although elevated concentrations of K in the root medium alters osmotic balance in plants, it was also demonstrated that 50 mM KCl treatments of drought stressed *Poa pratensis* promoted stomatal reopening and rapid resumption of photosynthesis during drought recovery [36].

The responses of Pn in plants exposed to salinity can involve multiple processes, including reductions in photosynthetic pigments, electron transport, and enzymatic reactions that are involved in photosynthesis, as well as reductions in CO_2_ uptake due to stomatal closure [37,38]. Leaf chlorophyll concentration can be an important biochemical indicator of salt tolerance in plants since it significantly contributes to Pn and plant growth responses under salinity conditions [39,40]. While the longer-term effects of salinity on Pn involve severe damage to the photosynthetic apparatus by salt accumulation in leaves [37,41], short-term effects have been often attributed to the reduced CO_2_ uptake due to stomatal closure [42]. In *Poa pratensis*, six days of treatments with all salts triggered large decreases in leaf chlorophyll concentrations, while relatively minor changes were observed in *Poa juncifolia* and more than two-fold increases in chlorophyll concentrations were measured in *Puccinellia nuttalliana* treated with 150 M NaCl and Na_2_SO_4_. Sodium appears to be a crucial element for chloroplast development and its function in halophytes and, unlike K, Na can increase both the number of chloroplasts and chlorophyll concentrations in the leaves of halophytic plants (Bose et al., 2017). In *Atriplex vesicaria*, a low concentration of Na triggered chlorosis even when the concentration of K was high [43]. In the isolated chloroplasts of halophytic quinoa (*Chenopodium quinoa*) and pigface (*Carpobrotus rosii*) plants, Pn could be maintained under high concentrations of Na (100 mM) and a low concentration of K (50 mM) [44]. It was also demonstrated that in the chloroplasts of halophytic plants, Na concentrations could be 20-times higher compared with the glycophytes [45].

In our study, the rapidity of the Pn responses to salts point to stomatal limitations as the key factors contributing to the Pn declines in *Poa pratensis* and *Poa juncifolia*. Leaf water potentials in both plant species sharply decreased when measured after six days of treatments with all salts, while in *Puccinellia nuttalliana*, leaf water potentials significantly decreased only in the 150 mM Na_2_SO_4_ treatment. The decreases in water potentials in *Poa pratensis* and *Poa juncifolia* are likely a combination of the reduced water uptake and the accumulation of salts and organic solutes in the leaf tissues resulting in decreased osmotic potentials. Glycophytes have limited ability to reduce the entry and accumulation of salts in roots or to exclude salts from the leaves [30]. Reductions in water uptake due to decreased osmotic potential of the root medium and decreased root hydraulic conductivity are major factors contributing to reduced water contents in salt-affected plants [46,47,48]. Similarly to stomatal regulation, root hydraulic conductivity is dynamically regulated and affected by various environmental factors, which may alter water delivery to the transpiring areas and upset water balance [49,50]. Studies on purified plasma membrane vesicles of *Beta vulgaris* demonstrated their high water permeability, suggesting that efficient cell-to-cell water transport under salt stress plays a significant role in water balance maintenance [51]. Enhanced cell hydraulic conductivity in NaCl-treated *Puccinellia nuttalliana* was also proposed to play a major role in the salt tolerance of this halophytic grass [20] and likely involves the reported increase in gene expression of the *PIP2;2* aquaporin [21].

Leaf water potentials and shoot water contents of *Puccinellia nuttalliana* were not altered by the 150 mM NaCl, demonstrating the ability of plants to maintain water balance. Salt exclusion, sequestration, and secretion, as well as the accumulation of organic solutes, are important mechanisms contributing to the maintenance of osmotic balance in halophytes [6]. Halophytic turfgrasses can exclude salt from the root cortex [52] and to secrete salt through the salt glands or salt bladders in the leaf epidermis [21,53]. Some halophytes also maintain osmotic potential by accumulating salt in the vacuoles [40]. The Na concentrations in roots of *Puccinellia nuttalliana* treated with NaCl and Na_2_SO_4_ increased to the similar levels as in the other two grass species. However, the shoot Na concentrations in these treatments in *Puccinellia nuttalliana* were only approximately one-half of the concentrations measured in *Poa pratensis* and *Poa juncifolia*, suggesting restricted root-to-shoot transport or (and) salt secretion. Extensive salt secretion through the leaves was observed in the earlier study in NaCl-treated *Puccinellia nuttalliana* [21]. Similarly to other plants [54], roots of the three studied grass species accumulated only a relatively small fraction of Cl compared with shoots, and there were no significant differences in Cl concentrations of roots and shoots between the species.

Contrary to *Poa pratensis*, in which root K concentrations decreased and shoot concentrations remained unchanged in the NaCl and Na_2_SO_4_ treatments, shoot and root K concentrations in *Puccinellia nuttalliana* increased in plants treated with Na salts. Interestingly, in the intermediate salt tolerant species, *Poa juncifolia*, the root and shoot K concentrations remained little changed compared with control plants. Salinity can disrupt the K balance in the cytosol and disrupt metabolic pathways since, in addition to its role in regulating osmotic balance, K is required for the activation of various cytosolic enzymes [55]. Strong correlations between the tissue K concentration and salt tolerance have been frequently reported for many plants [56,57,58]. High K:Na ratio is essential for maintaining cell metabolism, including protein biosynthesis [59]. A high K:Na ratio in mesophyll cells was suggested to be the principal factor contributing to salt tolerance in *Thellungiella halophila*, while the lack of this feature contributed to salt sensitivity in *Arabidopsis* [60]. Sustained root water uptake by the NaCl-treated halophytic grasses was also attributed to the maintenance of stable K levels in the roots [20], likely involving the high affinity K^+^ transporter PnHKT1;5, which was upregulated by NaCl in *Puccinellia nuttalliana* [21]. High salt concentrations trigger K efflux through the depolarization-activated outward-rectifying K channels [61]. The Na influx and K efflux cause activation of ATPase pumps and hyperpolarization of the membranes. As a result, K uptake is increased by the activation of two specific K channels, including voltage-dependent hyperpolarization-activated (KIR) and depolarization-activated (KOR) Shaker-type K channels [59]. The increased activity of H^+^ATAPase pumps can also provide a driving force for the activation of high affinity K transporters and increase K concentration during salinity [61].

The maintenance of water balance requires efficient water delivery to leaves. In the present study, both Na salts enhanced L_pc_ in *Puccinellia nuttalliana* by about two-fold compared with the untreated control, but decreased L_pc_ in *Poa juncifolia* and *Poa pratensis*. It is noteworthy that 150 mM KCl had no significant impact on L_pc_ in *Puccinellia nuttalliana* and triggered relatively minor decreases in L_pc_ in *Poa pratensis* and *Poa juncifolia*. An inhibition of root hydraulic conductivity is among the most sensitive initial responses of plants to salt stress [12,62] and involves rapid reductions in the aquaporin-mediated cell-to-cell water transport. In the wild-type *Arabidopsis*, NaCl concentration as low as 10 mM decreased L_pc_ by three-fold within 30 min following its application to roots and there was no effect on NaCl on L_pc_ in the AtPIP2;5 overexpression lines [16].

The results of our study clearly demonstrated that Na was the main factor contributing to the inhibition of L_pc_ in *Poa pratensis* and *Poa juncifolia* subjected to the Na salts and it was the factor responsible for the enhancement of L_pc_ in *Puccinellia nuttalliana*. Despite the differences in osmotic potentials between 150 mM Na_2_SO_4_ and 150 mM NaCl, their effects on L_pc_ in this halophytic grass were similar. Additionally, although osmotic potentials of 150 mM NaCl and 150 mM KCl are similar, the KCl treatment had no significant effect on L_pc_, while the NaCl treatment enhanced L_pc_ in *Puccinellia nuttalliana*. It also appears that despite the two-fold higher Na concentration in the Na_2_SO_4_ compared with NaCl treatment solutions, the effects of both salts on L_pc_ were similar, which could possibly be attributed to the contributions of the associated anions [11,63].

Although NaCl effects include direct ion toxicity and osmotic imbalance that can both contribute to root hydraulic conductivity reductions [19,64], the two stresses may vary in their modes of action on the aquaporin-mediated water transport [64]. Since the treatments with 50 µM HgCl_2_ brought the L_pc_ values in all three plant species and in all salt treatments to a similar level, the treatment effects can be attributed to the mercury-sensitive aquaporin-mediated water transport. Although HgCl_2_ is not a specific aquaporin inhibitor, in low concentrations, as used in our study, it inhibits the functionality of most of the aquaporins in the absence of other effects such as respiration [65,66]. Mercury blocks the central pore or through changes in the conformation of Ar/R region by attaching to the Cys residues [65,66]. Therefore, the enhancement of L_pc_ in *Puccinellia nuttalliana* by NaCl and Na_2_SO_4_ points to the effects of Na on the aquaporin-mediated water transport.

Aquaporins are proteins forming water channels in cell membranes to facilitate the transport of water and other small molecules including gases and some ions across the membranes [65,67,68,69]. Therefore, maintaining the functionality of aquaporins under salinity conditions could have important consequences to the transport of these molecules in halophytic plants. With typically between 30 and 70 aquaporin genes that are present in various plant species and many possible transcriptional and posttranscriptional regulations [13], the enhancement of aquaporin-mediated water transport in *Puccinellia nuttalliana* by Na may involve complex regulation mechanisms. Links between the inhibition of root water transport by NaCl and aquaporin function have been studied in various glycophytic plants and attributed to changes in the aquaporin abundance [70], gene expression [16,71,72], aquaporin phosphorylation and (or) dephosphorylation [15,16,19], membrane trafficking [73], pH, and Ca [15,51]. It appears that the strategy of *Puccinellia nuttalliana* plants to maintain water homeostasis in the presence of NaCl involves large increases in root transcript levels of the fast water transporting *PIP2;2* aquaporin, while decreasing gene expression levels of the tonoplast *TIP* aquaporins [21]. The increased *PIP2;2* gene expression could be a major factor contributing to the enhancement of cell hydraulic conductivity by the Na salts reported in the present study. We cannot reject the possibility that, in addition to its role as a fast water transporter (unpublished data), PIP2;2 could be involved in the transport of ions, including Na and K as reported for several plant PIP2s including PIP2;1 and PIP2;2 from *Arabidopsis thaliana* [74] and PIP2;8 from *Hordeum vulgare* [75]. However, this is not likely the case in our study since the cation conductance of these aquaporins is, to different degrees, blocked by Ca^2+^ [69], and we also found no significant differences in Na root concentrations between *Puccinellia nuttalliana* and the two other studied plants. The lower Na shoot concentrations in *Puccinellia nuttalliana* compared with the less salt-tolerant grasses were found to be facilitated by its secretion through salt glands present in the leaves, combined with enhanced cell wall lignification of the endodermis and xylem vessels [21], as also reported for other halophytes [76,77]. It was proposed that the increased transcript levels of several cyclic nucleotide-gated channels by NaCl in *Puccinellia nuttalliana* could be associated with the enhanced Na loading into the xylem as part of the salt tolerance mechanisms [21].

Low pH of the cytoplasm is among the common factors inhibiting aquaporin activity in plants subjected to environmental stresses [78]. There is mounting experimental evidence that salinity increases the apoplastic pH and decreases pH of the cytosol [79,80,81], which may be reversed by treatments of plants with Ca [80]. This may partly explain the alleviating effect of Ca on salt stress and aquaporin function in plants [80]. The observed increase in tissue K concentration in *Puccinellia nuttalliana* in this and the earlier study [20] could be expected to affect H^+^ fluxes, leading to an increase in cytosolic pH and an enhancement of aquaporin activity. Although the KCl treatment also enhanced the accumulation of K in roots in all three studied plant species, the presence of Na could differently affect K fluxes and its intracellular concentrations.

It is also noteworthy that, contrary to *Poa pratensis* and *Poa juncifolia*, in which salt treatments decreased root Ca concentrations and as opposed to the KCl treatment, which reduced root Ca concentrations in *Puccinellia nuttalliana*, the NaCl and Na_2_SO_4_ treatments increased root concentrations of Ca in this halophytic grass. It was reported that under salinity stress, the concentrations of Ca in halophytes can be several-fold higher compared with glycophytes, in which elevated salinity commonly inhibits Ca uptake by roots [82]. Calcium accumulates in Golgi and ER soon after the exposure of plants to salt stress and before it is transported to other organelles and the cytosol [83]. The increase in Ca in the cytoplasm of halophytes exposed to salt results in the activation of the salt overly sensitive (SOS) signaling pathway [84]. The SOS complex regulates specific transporters, including the NHX exchangers and Na^+^/H^+^ antiporters, to exclude Na from the cytosol and sequester it in vacuoles and intracellular spaces [82].

The present results, together with an earlier reported up-regulation of several SOS pathway genes in the NaCl-treated roots of *Puccinellia nuttalliana* [21], demonstrate that the maintenance of high root Ca concentrations in *Puccinellia nuttalliana* is likely an important factor contributing to its tolerance of the Na salts. However, they do not directly explain the enhancement of the aquaporin-mediated transport by the Na salts. High cytosolic Ca is commonly associated with aquaporin closure [15,51]. However, treatments of plants with Ca salts were also demonstrated to alleviate NaCl [15] and low root temperature [85] stresses by upregulating the activities of aquaporins. This enhancement was explained as a likely effect of Ca on the Ca-dependent phosphorylation and dephosphorylation [15,85]. The effect of Ca on maintaining cell membrane integrity under stress conditions [86] could also be an important factor supporting water transport activities. Clearly, the role of Ca in the regulation of the aquaporin-mediated water transport in plants exposed to salinity deserves further attention.

## 4. Materials and Methods

### 4.1. Plant Material and Treatments

Seeds of Kentucky bluegrass (*Poa pratensis* L.), alkali bluegrass (*Poa juncifolia* Scribn.), and Nuttall’s alkali bluegrass [*Puccinellia nuttalliana* (Schult.) Hitchc.] were collected in central Alberta, Canada. The seeds were surface sterilized with 70% ethanol for 2 min followed by 1% sodium hypochlorite for 5 min. Sterilized seeds were washed several times with the autoclaved distilled water and germinated on the sterile half-strength Murashige & Skoog (MS) solid medium without sucrose and hormones [87].

Several days after seed germination, the seedlings were transferred to 500 cm^3^ pots filled with commercial growing mix (Sunshine^®^ Mix #4 Professional Growing Mix, Sun Gro Horticulture, Seba Beach, AB, Canada). The plants were grown for 8 weeks in a controlled-environment growth room set to 22/18 °C (day/night) temperature, 65 ± 10% relative humidity, and 16 h photoperiod with 300 μmol m^−1^ s^−1^ photosynthetic photon flux density (PPFD) provided by the full-spectrum fluorescent bulbs (Philips high output, Markham, ON, Canada). They were fertilized weekly with half-strength modified Hoagland’s solution [88] and watered three times per week to runoff.

After 8 weeks of growth, seedlings were removed from the soil and placed in 12 L plastic containers filled with 50% Hoagland’s solution. Styrofoam boards were floated on the top of nutrient solution with holes cut in each board through which seedling roots were slipped into the solution and the stems secured to the board with foam stoppers. There were three repeated experiments for different sets of measurements. In each experiment, there were three plants per species randomly placed in each of the 12 replicated containers (three containers per treatment with nine plants in total in each container). The mineral solution was aerated with an air pump to provide O_2_ concentration of approximately 8 mg L^−1^. After 1 week of acclimation to hydroponic conditions, plants were treated with 150 mM NaCl, 150 mM Na_2_SO_4_, or 150 mM KCl for up to 10 days. Providing plants with the same salt concentrations enabled us to compare the osmotic and ionic effects of salts, as well as the effects of different Na^+^ concentrations in the presence of different anions (NaCl vs. Na_2_SO_4_). The salts were gradually added during the day (3 × 50 mM) to reduce osmotic shock. The control group consisted of plants growing in 50% Hoagland’s solution with no added salts. For all measurements, plants were randomly picked from the containers.

### 4.2. Plant Morphology and Dry Weights (DW)

After 10 days of treatments, plants were inspected for the visible impact of salts. Six plants from each treatment were harvested and their shoots and roots were separated. The shoots, and roots were placed in an oven at 70 °C for 72 h and weighed for the DW determinations.

### 4.3. Net Photosynthesis (Pn) and Transpiration (E) Rates

The measurements of Pn and E were carried out after 3, 6, and 9 days of treatments from approximately 5 to 9 h following the onset of photoperiod and by alternating plants from the different treatments. Three fully expanded uppermost leaves from each plant were marked and used for the measurements using a LI-6400 portable photosynthesis system with a 2 × 3 cm^2^ leaf chamber (LI-COR Biosciences, Lincoln, NB, USA). The reference CO_2_ concentration was 400 μmol mol^−1^, the flow rate was 200 μmol s^−1^, and the relative humidity (RH) level was set to 50% in the cuvette. The leaf chamber temperature was maintained at 20 °C, and PPFD was 400 μmol m^−2^ s^−1^ provided by the red-blue light spectrum of the light attachment. To determine leaf areas, the parts of the leaves that were inserted into the leaf chamber were excised after the last measurement and scanned. The leaf areas were calculated using the Sigmascan Pro 5.0 computer software (Systat Software, San Jose, CA, USA).

### 4.4. Leaf Chlorophyll Concentrations

Chlorophyll-a (chl-a), chlorophyll-b (chl-b) and total chlorophyll concentrations were determined in six randomly selected seedlings per treatment (*n* = 6) after 6 days of treatments. Fully expanded leaves were freeze-dried and grinded in a Thomas Wiley Mini-Mill (Thomas Scientific, Swedesboro, NJ, USA). Chlorophyll was extracted from the leaf samples (10 mg dry weight) with 8 mL dimethyl sulfoxide (DMSO) at 65 °C for 22 h. Chlorophyll concentrations were measured in DMSO extracts with a spectrophotometer (Genesys 10S-UV-VIS, Thomas Scientific, Swedesboro, NJ, USA) at 648 nm and 665 nm for chlorophyll-a and chlorophyll-b, respectively. Total chlorophyll concentrations (chlorophyll a + b) were calculated using the Arnon’s equation [89].

### 4.5. Leaf Water Potentials (ψ_w_) and Shoot Water Contents (WC)

The measurements of (ψ_w_) were carried out after 6days of treatments in six plants per treatment (*n* = 6) using a Scholander pressure chamber (PMS instruments, Corvallis, OR, USA) as previously described [20]. The measurements were carried out from approximately 5 to 9 h following the onset of the photoperiod. The same leaf that was marked and used for the gas exchange measurements was excised and immediately placed in a Scholander pressure chamber with the cut end of the leaf protruding through the lid. The chamber pressure was increased until xylem sap was released from the excised leaf and the balance pressure was recorded.

Shoot water contents were determined after 6 days of treatments in six plants per treatment (*n* = 6). The shoots were excised from each plant and weighed to establish their fresh weight (FW). They were then dried in an oven at 70 °C for 72 h and weighed to determine the dry weights (DW). The shoot WC was calculated using the following equation:

WC (%) = [(FW − DW)/FW] × 100

### 4.6. Cell Hydraulic Conductivity (L_pc_)

A cell pressure probe was used to determine L_pc_ of the root cortex cells in plants subjected to 6 days of treatments with 150 mM NaCl, 150 mM Na_2_SO_4_, 150 mM KCl, and in untreated control. Six plants per treatment were taken for the measurements (*n* = 6). The roots were placed on a metal sledge covered with a paper towel and the respective treatment solutions were flown along the roots. Micro capillaries used with the cell pressure probe were pulled to a fine point using a pipette puller (Kopf Vertical puller, Model 72, Tujunga, CA, USA) and subsequently ground to openings ranging from 8 to 10 µm. The micro capillaries were filled with silicone oil (Type AS4, Wacker, Munich, Germany). The tip of the micro-capillary was inserted 20 mm above the root apex into the cortical layer of plant roots. When the cell was punctured, half-time of water exchange (T_1/2_), turgor pressure (Pt), and cell elasticity (ε) were determined as earlier described [16,20] to calculate cell hydraulic conductivity. Once the hydraulic parameters were recorded, HgCl_2_ was added to the treatment solutions to the final concentration of 50 µM and the parameters recorded again [90]. Mercury inhibits aquaporin activity by selectively binding to Cys residues within the pore and HgCl_2_ has been commonly used in low concentrations to block water transport across aquaporins [65,66]. Following the measurements, thin sections of roots were examined under the microscope to determine cell dimensions and the cell volume for the L_pc_ calculations [90].

### 4.7. Tissue Elemental Analyses

The elemental analyses were carried out in the Natural Resources Analytical Laboratory of the University of Alberta, Edmonton, Canada. For the analyses, root and shoot samples (0.2 g dry weight) of six plants per species (*n* = 6) were collected after ten days of the different salt treatments. The roots were quickly rinsed in deionized water and blotted dry. To determine tissue concentrations of Na, K, and Ca, the samples were digested with 70% HNO_3_ and heated for 10 min at 185 °C in a microwave oven (Mars 5 Microwave Accelerated Reaction System, CEM, Matthews, NC, USA). The extracts were diluted with Milli-Q water, filtered, and analyzed by with the inductively coupled plasma—optical emission spectrometer (iCap 6000, Thermo Fisher Scientific Inc., Waltham, MA, USA). Tissue Cl was analyzed in hot water extracts using the EPA 325.2 ferric thiocyanate method (US Environmental Protection Agency 1983) with the Thermo Gallery Plus Beermaster Autoanalyzer (Thermo Fisher Scientific, Vantaa, Finland).

### 4.8. Statistical Analyses

Statistical analyses were carried out using the SPSS 18.0 statistical package (SPSS Inc., Chicago, IL, USA). One-way ANOVA was carried out followed by the Tukey’s test to detect significant differences between the treatments for each plant species (*p* ≤ 0.05). The L_pc_ data were analyzed for significant differences between the treatments and species as the main factors. The data for each measurement were obtained from the same experiment with each replicate representing one plant. The data that did not meet the ANOVA assumptions of normality of distribution and homogeneity of variance were transformed with a log10 function.

## 5. Conclusions

Our study demonstrated that sustaining growth, chlorophyll concentrations, gas exchange, and water transport in *Puccinellia nuttalliana* requires the presence of Na in the applied salt treatments. The maintenance of high Pn in this halophytic grass could be explained by a combination of stomatal and non-stomatal factors, including leaf chlorophyll concentrations. The enhanced L_pc_ in *Puccinellia nuttalliana* by Na (150 mM NaCl and 150 mM Na_2_SO_4_) treatments was due to the mercury-sensitive aquaporin-mediated water transport that could reflect the earlier reported enhancement of gene expression of some of the PIP2 aquaporins by NaCl in *Puccinellia nuttalliana*. Ca and K accumulation in roots that was triggered by Na likely played a role in regulating the aquaporin gating properties and (or) aquaporin gene activity in this halophytic plant.

## Data Availability

The data are available upon request.

## Supplementary Materials

The following supporting information can be downloaded at: https://www.mdpi.com/article/10.3390/ijms23105732/s1.
