# Supplementary figures and images for: Salinity Tolerance of Halophytic Grass Puccinellia nuttalliana Is Associated with Enhancement of Aquaporin-Mediated Water Transport by Sodium
Source: Int J Mol Sci. 2022 May 20;23(10):5732. doi: 10.3390/ijms23105732 (PMC9145133; doi:10.3390/ijms23105732)

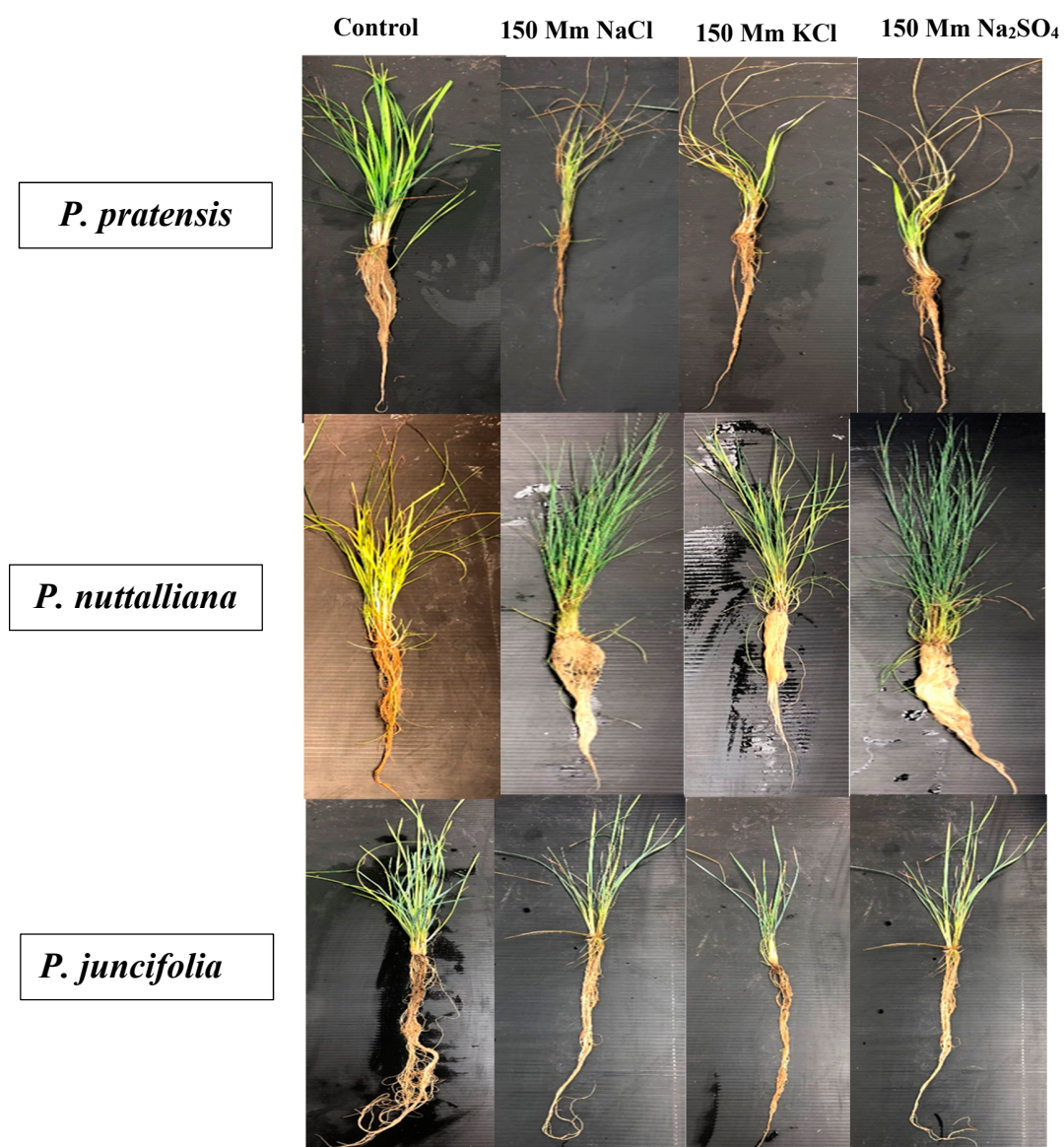

Supplementary Figure S1

Supplement: Supplementary file 1 [file ijms-23-05732-s001.zip › ijms-1687597-supplementary.pdf]
